# Association between the temperature difference and acute exacerbations of chronic obstructive pulmonary disease: A time-series analysis with 143,318 hospital admissions in Beijing, China

**DOI:** 10.3389/fpubh.2023.1112926

**Published:** 2023-01-26

**Authors:** Jia Fu, Yanbo Liu, Yakun Zhao, Yuxiong Chen, Zhenge Chang, Kai-Feng Xu, Cheng Huang, Zhongjie Fan

**Affiliations:** ^1^Department of Cardiology, Peking Union Medical College Hospital, Peking Union Medical College, Chinese Academy of Medical Sciences, Beijing, China; ^2^Department of International Medical Services, Peking Union Medical College Hospital, Peking Union Medical College, Chinese Academy of Medical Sciences, Beijing, China; ^3^Department of Respiratory Medicine, Peking Union Medical College Hospital, Peking Union Medical College, Chinese Academy of Medical Sciences, Beijing, China; ^4^Department of Thoracic Surgery, Peking Union Medical College Hospital, Peking Union Medical College, Chinese Academy of Medical Sciences, Beijing, China

**Keywords:** AECOPD, temperature change, temperature, temperature difference, distributed lag non-linear model

## Abstract

**Purpose:**

Acute exacerbation of chronic obstructive pulmonary disease (AECOPD) has the adverse influence on quality of life and creates significant healthcare costs. However, there were sparse studies investigating the correlation between AECOPD hospital admissions and temperature change. Therefore, it is noteworthy to investigate the impact of various temperature differences and recognize the susceptible population. The purpose of this study was to investigate the impact of temperature differences on AECOPD hospital admissions, and to give potentially helpful material for disease preventative efforts.

**Methods:**

The distributed lag non-linear model was adopted to characterize the exposure-response relationship and to assess the impact of temperature difference. The stratified analysis and sensitivity analysis were also conducted to determine the susceptible populations and examine the robustness of the results.

**Results:**

There were 143,318 AECOPD hospital admissions overall during the study period. The AECOPD hospital admissions had significant association with the daily mean temperature difference (DTDmean) such as the extreme-cold temperature difference (1st DTDmean), the ultra-cold temperature difference (5th DTDmean), the ultra-hot temperature difference (95th DTDmean) and the extreme-hot temperature difference (99th DTDmean). Besides, there was the “*U*-shaped” association between DTDmean and 21 days cumulative relative risk of AECOPD.

**Conclusion:**

The AECOPD hospital admissions was correlated with the DTDmean temperature differences, especially the extreme-cold and extreme-hot temperature difference. Moreover, people older than 65 years were more susceptible to the extreme-hot and extreme-cold temperature difference.

## 1. Introduction

Chronic obstructive pulmonary disease (COPD) is a general disease characterized by ongoing airflow restriction and respiratory symptoms (1). Globally, COPD is a leading cause of morbidity and mortality (2). Statistics show that COPD is now one of the three leading causes of death worldwide, with 90% of deaths taking place in low- and middle-income nations (3). A thorough investigation found that COPD contributed 2.6% of the world's disability-adjusted life years in 2015 (4). Because of the ongoing exposure to relevant risk factors and an aging population, it is anticipated that the burden of COPD will rise globally in the future decades (5). Acute worsening of the initial respiratory symptoms that necessitates a change in treatment is known as the acute exacerbation of COPD (AECOPD) (6). According to estimates, COPD patients experience 1–4 exacerbations annually (7). AECOPD might hasten the deterioration of lung function (8), which lead to less physical activity and an increased risk of death (9, 10). AECOPD also has significant association with damaged quality of life (11), which are the major culprits of hospitalizations (12). Research has shown that AECOPD is to blame for 10% of all medical hospitalizations (13). In brief, AECOPD can generate huge healthcare burdens.

Previously reported works have demonstrated that ambient temperature have significant association with COPD hospitalization, emergency visits and so forth. For example, a Spanish study found that hospital admissions and mortality for COPD exacerbation were more frequent in autumn and winter (14). A case-crossover study in Taiwan demonstrated the exacerbation rate of COPD on event days increased by 0.8% for every 1°C decrease in air temperature (95% C.I. 1.015–1.138) (15). A study from 2004 to 2011 discovered that during periods of extreme weather, adults over 60 years old had an increased risk of admission for pneumonia and COPD (16). However, the impacts of ambient temperature have received more research attention. Studies on the relationship between AECOPD hospital admissions and temperature changes were scarce, particularly in Beijing, China. Additionally, the indicator of temperature change was relatively 1-fold. Hence, it is important to investigate the impact of various temperature differences and recognize the susceptible population. The purpose of this study was to investigate the impact of temperature differences on AECOPD hospital admissions, and to give potentially helpful material for disease preventative efforts.

## 2. Materials and methods

### 2.1. Data gathering

The hospital admissions data of the AECOPD were obtained from the Beijing Municipal Health Commission Information Center and this study was set as the period from January 1, 2013 to December 31, 2016. The data included the anonymous personal information (age, gender, discharge diagnosis, date of birth), hospital admission data (hospitalization date and hospital name), and address information (birthplace and present address). International Classification of Diseases 10th revision was used to define AECOPD (ICD-10: J41-J44). To rule out the effect of the floating population on the study's findings, the Beijing resident population were filtrated according to the address information of patients. The daily hospital admissions of different gender subgroups (male and female), different age subgroups (age <65, age ≥ 65) and total population were calculated by using the IBM SPSS 26 and R (version 4.1.1). The Peking Union Medical College Hospital (PUMCH) Institutional Review Board approved the study's conduct.

The China Meteorological Administration provided the daily meteorological data. It contained both the temperature data [daily maximum temperature (Tmax), mean temperature (Tmean), and minimum temperature (Tmin)], and other meteorological factors [Wind Speed (WS), Relative Humidity (RH), and Air Pressure (AP)]. As for meteorological data, four kinds of temperature differences were calculated respectively to represent the temperature change. It contained overall temperature range (TR, Tmax today minus Tmin today), daily mean temperature difference (DTDmean, Tmean today minus Tmean yesterday), daily maximum temperature difference (DTDmax, Tmax today minus Tmax yesterday), and daily minimum temperature difference (DTDmin, Tmin today minus Tmin yesterday). The level of air pollution was regarded as one of the confounding factors and adjusted in our study because prior research indicated that air pollution had an adverse influence on multiple respiratory disorders (17–19). Our study adjusted the Air Quality Index (AQI), which represented the main effects of the level of air pollution because there were numerous types of air pollutants and the daily major pollutant concentration levels fluctuated. The Beijing Environmental Protection Bureau provided the AQI data for the same period.

The influenza viruses can trigger exacerbations of respiratory diseases (20–22), the public holiday (PH) and day of the week (DOW) might have an influence on the behavioral patterns, so the influenza epidemic (IF), PH, and DOW were also regarded as confounding factors and adjusted in our statistical model. When the positive rate of influenza virus isolation in any given week exceeded 20% of the highest weekly positive rate in the observation season in the north of China, it was de-fined as the influenza endemic according to the method used in prior study (23). The Chinese National Influenza Center provided the influenza data (https://ivdc.chinacdc.cn/cnic/zyzx/lgzb).

### 2.2. Statistical analysis

Since temperature typically has a lag effect and prior research disclosed that correlation between COPD and temperature was non-linear (24, 25). The distributed lag non-linear model (DLNM) was constructed to concurrently match the effect and lag effect of independent variables (26). The distributed lag non-linear model was adopted to characterize the exposure-response relationship and to assess the impact of meteorological parameters (27). Because hospital admissions for respiratory disorders are small probability events that occur independently, the daily counts of AECOPD admissions were viewed as a quasi-Poisson distribution in this model to control the overdispersion in the number of hospitalizations (28). To eliminate the seasonal and long-term trends in AECOPD admission, the natural cubic spline function for time was applied. To eliminate the effects of other meteorological conditions and air pollution, the natural cubic spline functions for RH, WS, AP, and AQI were also included. As previously stated, the DOW, PH, and IF were also regarded as confounding factors and adjusted in model in the meantime. The main model is shown in detail below:


$$\begin{array}{l} \log\left[E\left(Y_{t}\right)\right]=\alpha +cb\left(Temp_{t},\;lag,df=4\right)\\ +ns\left(Time,\;df=7\;per\;year\right)+ns\left(WS,\;df=3\right)\\ +ns\left(AP,df=3\right)+\;ns\left(RH,df=3\right)\\ +\;ns\left(AQI,df=3\right)+\beta_{1}*DOW+\beta_{2}*IF+\beta_{3}*PH \end{array}$$

*E*(*Y*_*t*_) represent the number of AECOPD hospital admissions on day *t*. α and β were the model intercept and regression coefficient, respectively. *cb* meant the cross-basis function and *ns* indicates the natural cubic spline function. *Tmp* refer to the temperature difference. *Time* refers to the time to control the season and long-term trends. *WS, AP, RH*, and *AQI* represent the daily mean wind speed, air pressure, relative humidity, and air quality index on day *t*, respectively. *DOW, IF, and PH* are the indicator for day of the week, influenza epidemic status and public holiday. *df* represent the degree of freedom. All statistical analyses were performed using R (version 4.1.1) software, and the two-sided *p*-value of 0.05 was conducted to assess statistical significance.

The single day lag effect was examined using a 3D map that included lag days (Lag), relative risk (RR), and temperature differences. The maximum lag of 37 days was chosen to fully detect the influence of temperature differences and the probable harvesting effects. The figure of association between the specific percentile temperature difference (1st: extreme-cold temperature difference; 5th: ultra-cold temperature difference; 95th: ultra-hot temperature difference; 99th: extreme-hot temperature difference) and relative risk in various lag days were also charted. The extreme-cold, ultra-cold, ultra-hot and extreme-hot temperature difference means first, fifth, ninety-fifth and ninety-ninth percentile temperature difference. The cumulative relative risk of AECOPD hospital admission (CRR) was set to 21 days to fully detect the influence of temperature differences and avoid excessive accumulation. In addition, the stratified analysis was also conducted to determine the susceptible populations and examine the robustness of the results. In various subgroups, the most moderate temperature difference (MMTD) in the total population was taken as the standard. To assess the reliability of findings and the robustness of statistical model, we also conducted sensitivity analyses. We changed the degrees of freedom to 6 per year for *Time* and 4 for *RH, WS, AP*, and *AQI*, or 8 per year for *Time* and 5 for *RH, WS, AP*, and *AQI* in the statistical model to recalculate the CRR of temperature differences. R (version 4.1.1) was used for all statistical analysis along with the packages of “dlnm” and “mgcv.”

## 3. Results

### 3.1. Descriptive analysis

Tables 1, 2 summarized daily hospital admissions of AECOPD and meteorological data. The total population and subgroups' daily hospital admissions for AECOPD were shown in Table 1. There were 143,318 AECOPD hospital admissions overall between January 1, 2013, and December 31, 2016. While 24,938 of them were under 65 and 95,555 of them were men. There were 523 days of the influenza epidemic and 462 days of public holidays throughout the research period. The meteorological information is displayed in Table 2. The average values of Tmax, Tmean, Tmin, WS, RH, and AP were 18.95, 12.88°C, and 7.13°C, 9.29 m/s, 53.43% and 1,016.56 hPa respectively during the period of study. The average value of temperature range was 11.82°C and the average value of daily maximum/ mean/minimum temperature differences were all 0°C.

**Table 1:** AECOPD daily hospital admissions of the total population and subgroups in Beijing from January 1, 2013 to December 31, 2016.

	**Sum**	**Max**	**Min**	**Average**	**SD**
Total	143,318 (100%)	226	17	98.1	39.421
Male	95,555 (66.7%)	158	12	65.4	25.627
Female	47,763 (33.3%)	110	2	32.69	15.948
<65	24,938 (17.4%)	47	0	17.07	8.231
≥65	118,380 (82.6%)	189	13	81.03	32.678
IF	-	1 (*N* = 523)	0 (*N* = 938)	-	-
PH	-	1 (*N* = 462)	0 (*N* = 999)	-	-

SD, standard deviation; <65, people under the age of 65; ≥65, people at the age of 65 and above; IF, influenza epidemic (one represents the presence of influenza epidemic on the observation day); PH, public holiday (one represents the presence of public holiday on the observation day).

**Table 2:** Baseline information for different temperatures and meteorological data from January 1, 2013 to December 31, 2016.

	**Min**	**Max**	**Average**	**SD**
AQI	23	485	123.65	75.173
RH (%)	8	97	53.43	19.858
WS (m/s)	3	34	9.29	4.754
AP (hPa)	994	1,044	1,016.56	10.166
Tmean (°C)	−16	32	12.88	11.169
Tmax (°C)	−13	42	18.95	11.392
Tmin (°C)	−17	27	7.13	11.338
TR (°C)	1	26	11.82	4.31
DTDmean (°C)	−7	7	0	2.263
DTDmin (°C)	−9	11	0	2.778
DTDmax (°C)	−14	16	0	3.574

SD, standard deviation; AQI, air quality index; RH, relative humidity; WS, wind speed; AP, air pressure; Tmean_/_max_/_min, daily mean_/_max_/_min temperature; TR, temperature range; DTDmean_/_max_/_min, daily mean_/_max_/_min temperature difference.

### 3.2. Single day relative risk

**Figure 1:**
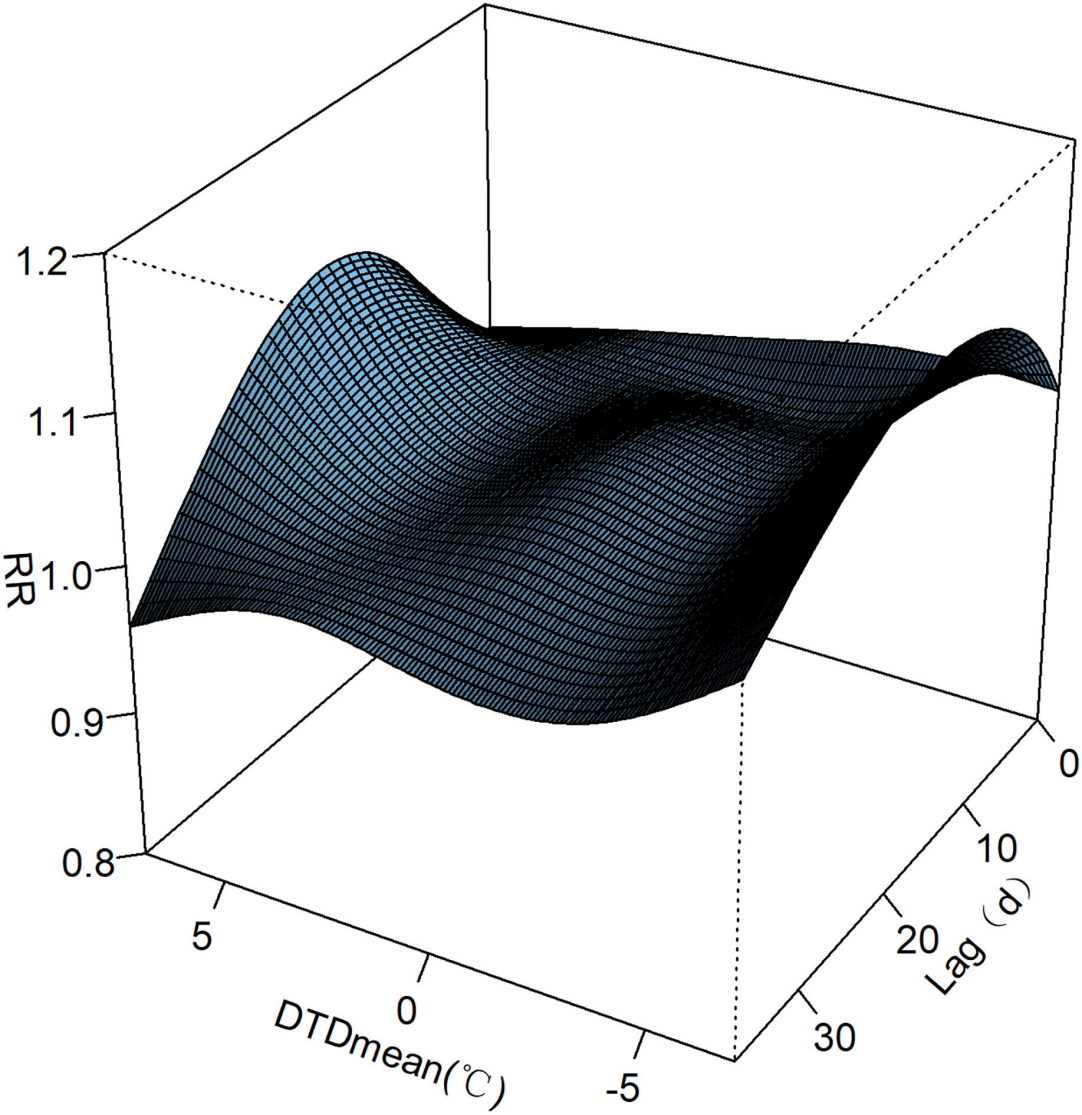
Association among the DTDmean, lag days and the relative risk of AECOPD hospital admissions in Beijing.

Figure 1 depicted the overall trend of the association among the DTDmean, lag days and the relative risk of AECOPD hospital admissions. In general, the RR became larger and larger with the increase of the absolute value of DTDmean in the mid-term of lag days.

**Figure 2:**
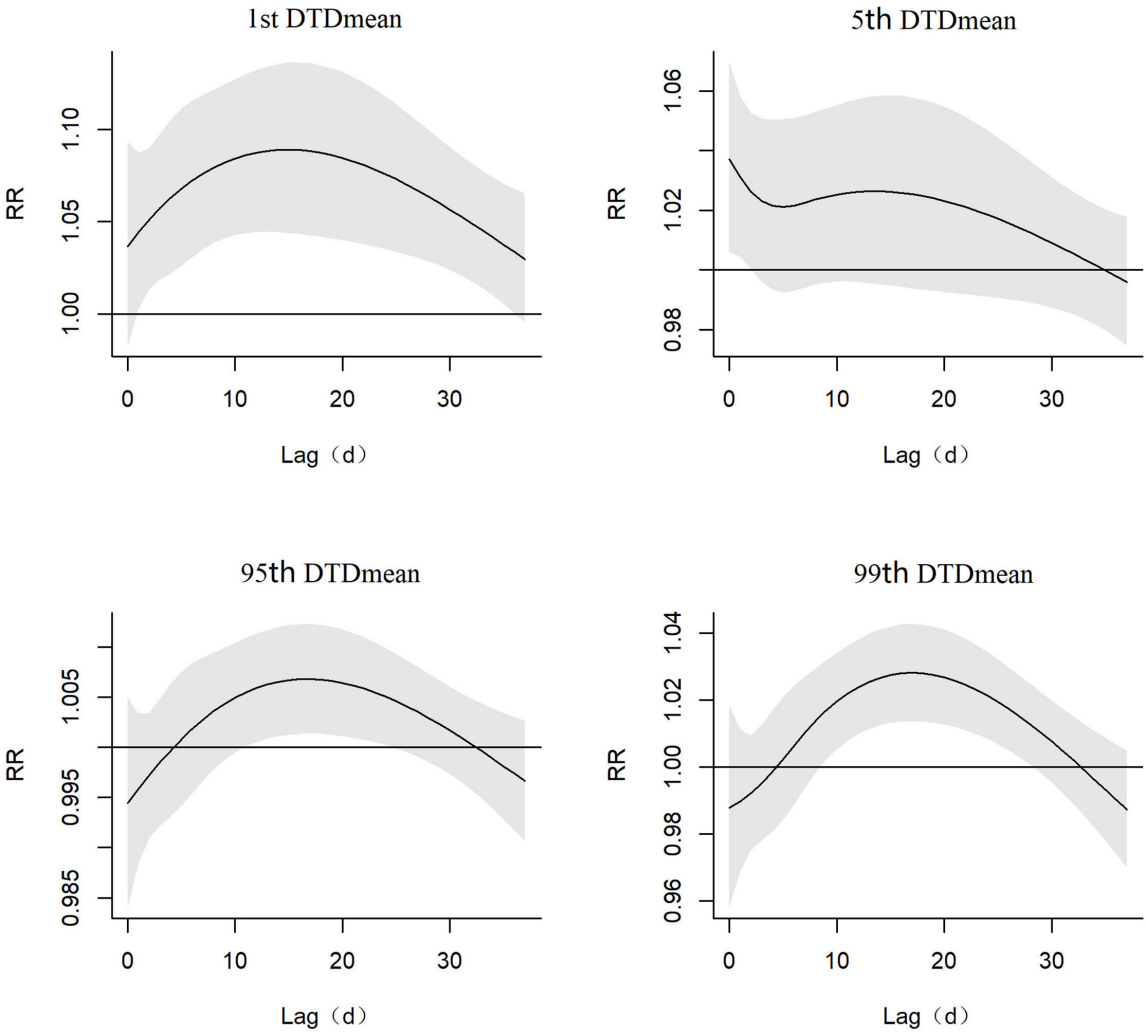
Relative risk (include 95% C.I.) of the extreme-cold, ultra-cold, ultra-hot, and extreme-hot of DTDmean temperature difference in AECOPD hospital admissions in different lag days.

Figure 2 depicted the result that the AECOPD hospital admissions had significant association with the extreme-cold temperature difference (1st DTDmean), the ultra-cold temperature difference (5th DTDmean), the ultra-hot temperature difference (95th DTDmean) and the extreme-hot temperature difference (99th DTDmean). As for the extreme-cold temperature difference, the AECOPD hospital admissions had significant association from lag 1 to lag 36 days and the maximum of RR was in lag 15 days. As for the ultra-cold temperature difference, the AECOPD hospital admissions had significant association from lag 0 to lag 2 days and the maximum of RR was in lag 0 days. As for the ultra-hot temperature difference, the AECOPD hospital admissions had significant association from lag 10 to lag 25 days and the maximum of RR was in lag 17 days. As for the extreme-hot temperature difference, AECOPD hospital admissions had significant association from lag 8 to lag 29 days and the maximum of RR was in lag 17 days.

### 3.3. Cumulated relative risk

**Figure 3:**
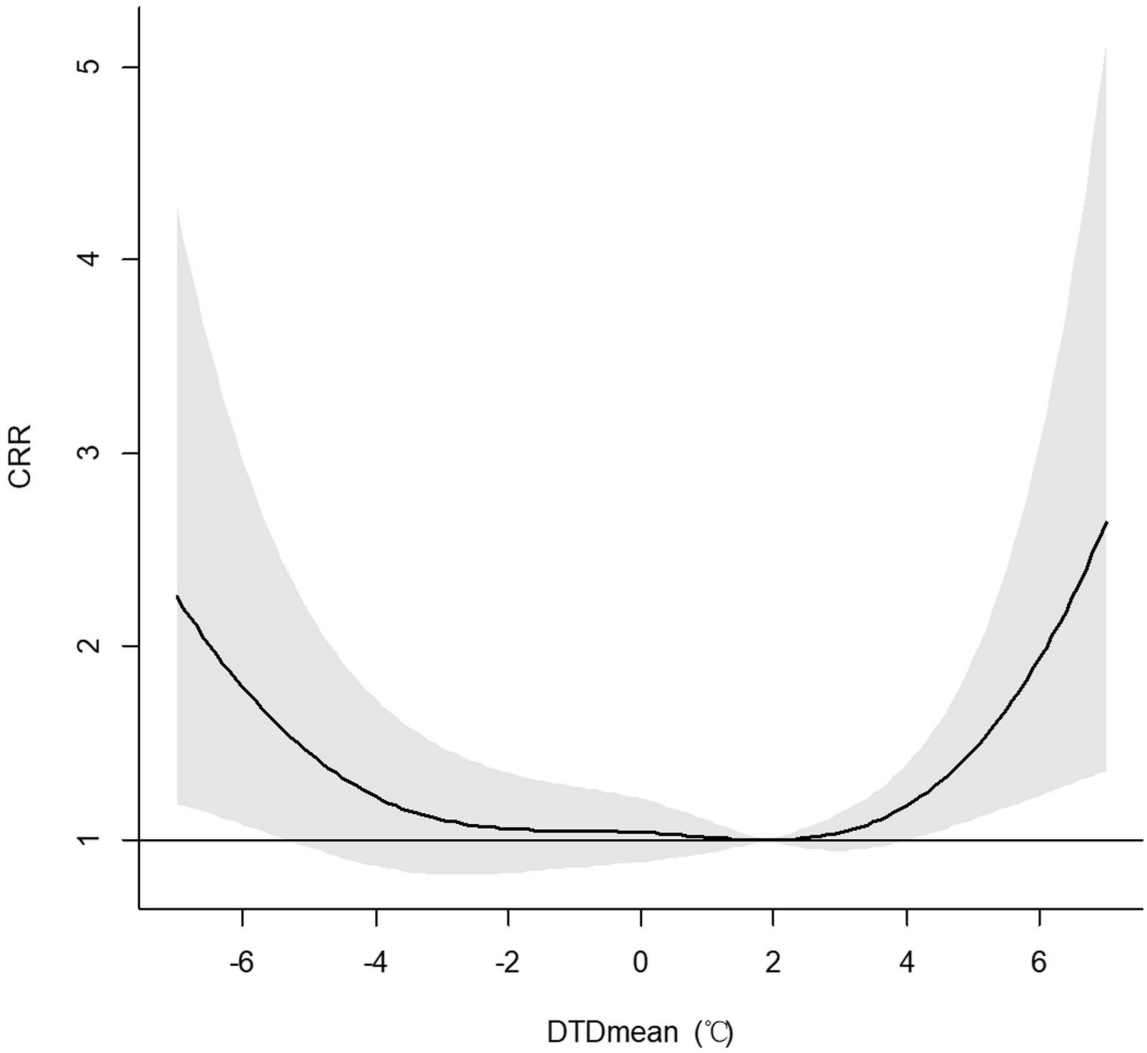
Association between the DTDmean and the 21 days cumulated relative risk (include 95% C.I.) of AECOPD hospital admissions among the total population in Beijing.

Figure 3 showed the *U*-shaped curve association between the DTDmean and the 21 days CRR (95% C.I.) of AECOPD hospital admissions. From Table 3, we found that the 21 days CRR of AECOPD was significant when it was in the extreme-cold temperature difference (1st DTDmean), the ultra-hot temperature difference (95th DTDmean) and the extreme-hot temperature difference (99th DTDmean). When the DTDmean was −6°C, the 21 days cumulated relative risk was 1.79 (95% C.I. 1.08–2.96) and when the DTDmean was 5°C, the 21 days cumulated relative risk was 1.46 (95% C.I. 1.11–1.93).

**Table 3:** The 21 days CRR and 95% C.I. of AECOPD in the total population in Beijing at the different DTDmean percentiles during the period from 2013 to 2016.

**Percentiles**	**CRR**	**95% C.I. low**	**95% C.I. upp**
P1 (−6°C)	1.79	1.08	2.96
P5 (−4°C)	1.22	0.87	1.73
P95 (4°C)	1.18	1.00	1.39
P99 (5°C)	1.46	1.11	1.93

CRR, cumulated relative risk; C.I., confidence interval.

### 3.4. Stratification effect

**Figure 4:**
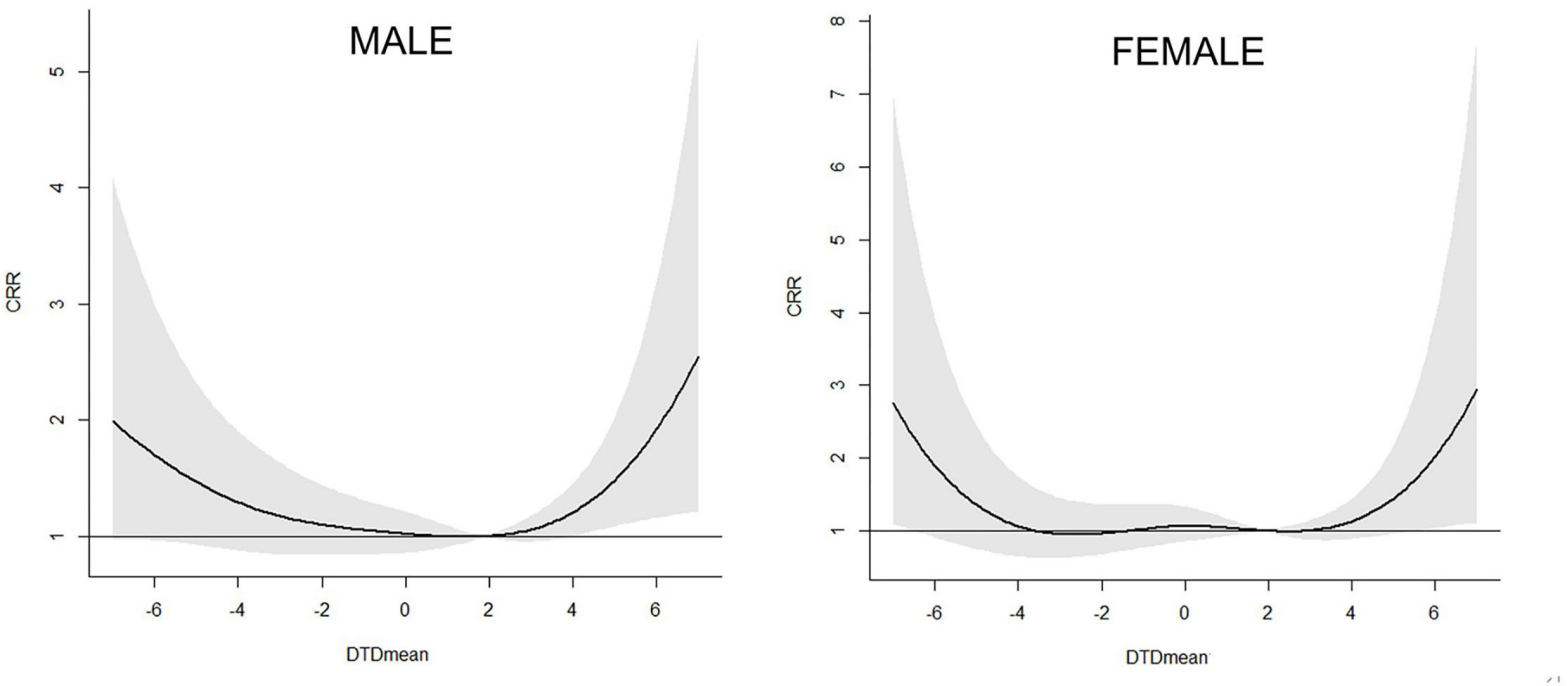
Association between the DTDmean and the 21 days cumulated relative risk (include 95% C.I.) of AECOPD hospital admissions in different gender subgroups in Beijing.

Figure 4 and Table 4 depicted the results for the association between the DTDmean and the 21 days CRR of AECOPD hospital admissions in different gender groups in Beijing. It showed that the effect of DTDmean was similar in male and female population. For example, when the DTDmean was −6°C (1st DTDmean), the 21 days CRR for male and female subgroups were 1.70 (95% C.I. 0.96–3.00) and 1.89 (95% C.I. 0.91–3.92), respectively.

**Table 4:** The 21 days CRR and 95% C.I. of AECOPD in the different sex groups in Beijing at the different DTDmean percentiles during the period from 2013 to 2016.

**Percentiles**	**Male (*****N*** = **95,555)**			**Female (*****N*** = **47,763)**		
	**CRR**	**95% C.I. low**	**95% C.I. upp**	**CRR**	**95% C.I. low**	**95% C.I. upp**
P1 (−6°C)	1.70	0.96	3.00	1.89	0.91	3.92
P5 (−4°C)	1.30	0.88	1.91	1.06	0.65	1.75
P95 (4°C)	1.21	1.00	1.45	1.13	0.89	1.44
P99 (5°C)	1.49	1.09	2.03	1.44	0.96	2.18

CRR, cumulated relative risk; C.I., confidence interval.

**Figure 5:**
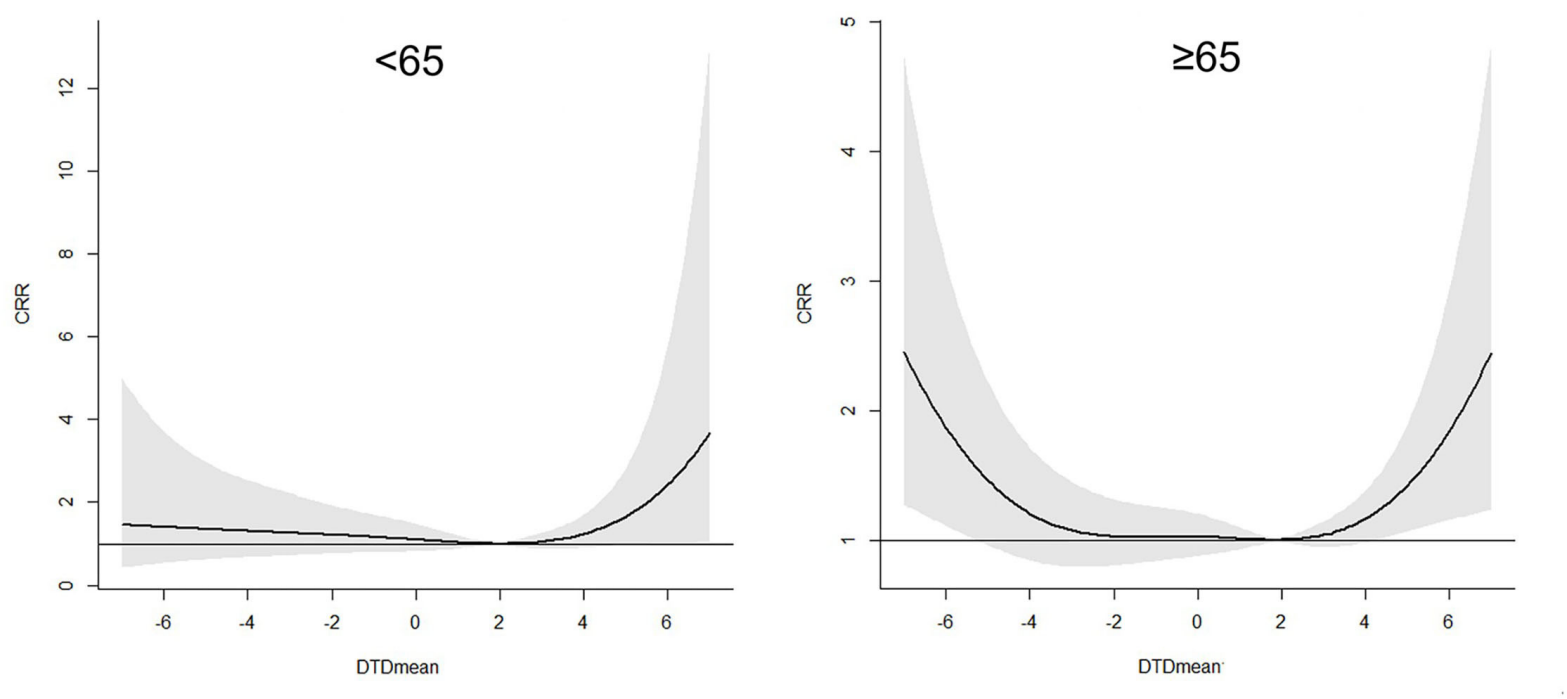
Association between the DTDmean and the 21 days cumulated relative risk (include 95% C.I.) of AECOPD hospital admissions in different age subgroups in Beijing.

Figure 5 and Table 5 depicted the results for the association between the DTDmean and the 21 days CRR of AECOPD hospital admissions among different age groups in Beijing. It showed that people older than 65 years were more susceptible to the extreme-hot and extreme-cold temperature difference. For example, when the DTDmean was −6°C (1st DTDmean), the 21 days CRR for younger and older subgroups were 1.41 (95% C.I. 0.53–3.37) and 1.87 (95% C.I. 1.11–3.14), respectively.

**Table 5:** The 21 days CRR and 95% C.I. of AECOPD in the different age groups in Beijing at the different DTDmean percentiles during the period from 2013 to 2016.

**Percentiles**	<**65 (*****N*** = **24,938)**			≥**65 (*****N*** = **118,380)**		
	**CRR**	**95% C.I. low**	**95% C.I. upp**	**CRR**	**95% C.I. low**	**95% C.I. upp**
P1 (−6°C)	1.41	0.53	3.37	1.87	1.11	3.14
P5 (−4°C)	1.31	0.67	2.54	1.20	0.84	1.72
P95 (4°C)	1.24	0.90	1.70	1.16	0.98	1.38
P99 (5°C)	1.66	0.97	2.83	1.42	1.07	1.89

CRR, cumulated relative risk; C.I., confidence interval; <65, people under the age of 65; ≥65, people at the age of 65 and above.

### 3.5. Other temperature differences

Additionally, we tried to look for the correlation between other temperature changes and AECOPD hospital admissions. However, most of them had no significance. Check out the Supplementary material for further information.

### 3.6. Sensitivity analysis

To examine how robust our results were, the degrees of freedom were modified. As shown in Supplementary Figures 7, 8, the results were in line with the original statistical model substantially. It showed that the primary models generated reliable findings.

## 4. Discussion

Utilizing a distributed lag non-linear model, our study discovered that there were significant correlations between the hospital admissions of AECOPD and the temperature difference. Overall, a large change in temperature could lead to a rise in AECOPD hospital admissions both when temperature raised and declined extremely.

Although different studies have different indicators of temperature change, most of the results were similar from different geological areas. For example, wintertime temperature variability was linked to a higher occurrence of incident respiratory disorders, according to a Chinese cohort of 66,820 older individuals in Hong Kong (≥65 years) with 10–13 years of follow-up. The HR per 1°C change in temperature variability (TV) was 1.20 (1.08–1.32) for all incident respiratory disorders and 1.41 (1.15–1.71) for COPD in wintertime (29). A Taiwan study showed that when the diurnal temperature range exceeded 9.6°C, COPD admissions to the emergency room rose by 14% (30). A Korean study found that daily temperature fluctuation was typically linked to emergency hospitalization for pneumonia, COPD and total respiratory. (31). Extreme temperature changes between adjacent days were linked to increased mortality for diseases like pneumonia, and COPD, according to a nationwide study that adopted mortality data from more than 100 cities in the USA (32). The study across 135 US cities found that the standard deviation of temperature was related to increases in the incidence of COPD mortality in yearly summer from 1985 to 2006 (33).

Several hypotheses can be made with care, even if the precise physiological processes of the temperature change effect on AECOPD are yet not completely understood. People's capacity to adjust to local climates was hampered by temperature variations, which could raise the chance of unfavorable health consequences like respiratory disorders (34, 35). Temperature changes have been proven to alter the physiological changes in the circulatory system, lower the immune system's response to respiratory infections, and create an inflammatory nasal response in people with persistent allergic rhinitis (36–39). All of these may trigger respiratory events. The effect also could be explained by the influence of extreme ambient temperatures in some way. The effects of temperature on the respiratory system were as follows: Patients with COPD may experience bronchoconstriction and inflammation due to the direct impacts of cold air, which became more pronounced as the temperature dropped (40). Cold temperature can also aggravate respiratory diseases by a rise in airway bacterial and viral infections, inflammatory factor infiltration, and mucus secretion that follows (41, 42). Cold temperature can affect mucin secretion through a variety of mechanisms, while abnormal airway mucin secretion can lead to obstruction of mucin clearance, increase the chance of infection, and contribute to the development of COPD (43). While cytokines may be released in response to a heated environment, this could lead to the inflammatory response and respiratory distress (44, 45).

In addition, our findings showed people older than 65 years were more sensitive to the extreme-cold or extreme-hot temperature difference. It was agreed with the results in previous studies. A nationwide study suggested that people over 75 and those with respiratory conditions were shown to be particularly vulnerable to temperature changes between days (32). Aging decreased thermoregulation ability and immune system responses to environmental factors (36, 46).

The study contains some solid points. First, the study collected information of over 143,000 AECOPD hospital admissions between January 1, 2013, and December 31, 2016. And the data covered all secondary and tertiary hospitals in Beijing. Longer study periods and larger sample sizes may yield more trustworthy and precise research findings. Furthermore, this is the first research on the association between temperature differences and hospital admissions for AECOPD in Beijing as far as we are aware. At the same time, we analyzed the various temperature differences to find the better indicators of temperature change. Lastly, a number of confounding variables were taken into account in the research, including the impact of the influenza, AQI, and statutory holiday. There are still some limitations to this study. Our research was limited to Beijing, thus results may not generalize to other locales. Besides, as the lack of individualized exposure data, many potential risk factors that could affect the AECOPD hospital admissions such as complication and medication situation were not included.

## 5. Conclusion

The AECOPD hospital admissions was correlated with the DTDmean temperature differences, especially the extreme-cold and extreme-hot temperature difference. Moreover, people older than 65 years were more susceptible to the extreme-hot and extreme-cold temperature difference.

## Data availability statement

The datasets presented in this article are not readily available because this dataset is now confidential. Requests to access these datasets should be directed to fanzhongjie@pumch.cn.

## Ethics statement

The studies involving human participants were reviewed and approved by Peking Union Medical College Hospital (PUMCH) Institutional Review Board. Written informed consent for participation was not required for this study in accordance with the national legislation and the institutional requirements.

## Author contributions

The conception and design in the study were contributed by JF, CH, and ZF. The database was organized by YC, JF, and YL. The methodology was provided by ZF. The statistical analysis was done by ZC and YZ. The software was operated by K-FX and CH. The first draft of the manuscript was written by JF. The submitted version of the work was reviewed, revised, and approved by all authors.
