# Supplementary material for: Association between the temperature difference and acute exacerbations of chronic obstructive pulmonary disease: A time-series analysis with 143,318 hospital admissions in Beijing, China
Source: Front Public Health. 2023 Jan 26;11:1112926. doi: 10.3389/fpubh.2023.1112926 (PMC9909227; doi:10.3389/fpubh.2023.1112926)

Figure S1 The temperature range's (1st 5th 10th and 25th TR) relative risk (include 95% C.I.) of AECOPD hospital admissions in the total population in different lag days

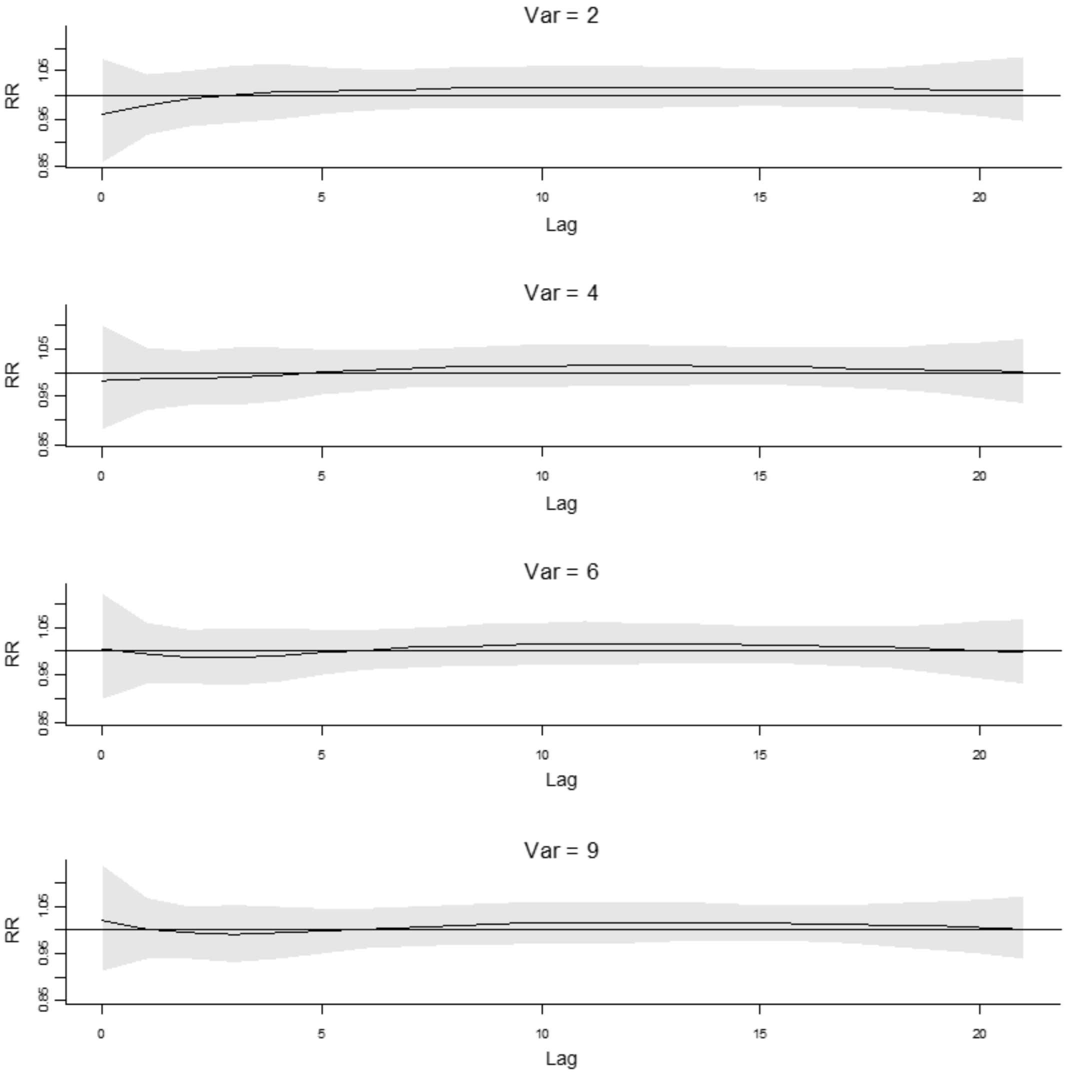

Figure S2 The temperature range's (75th 90th 95th and 99th TR) relative risk (include 95% C.I.) of AECOPD hospital admissions in the total population in different lag days

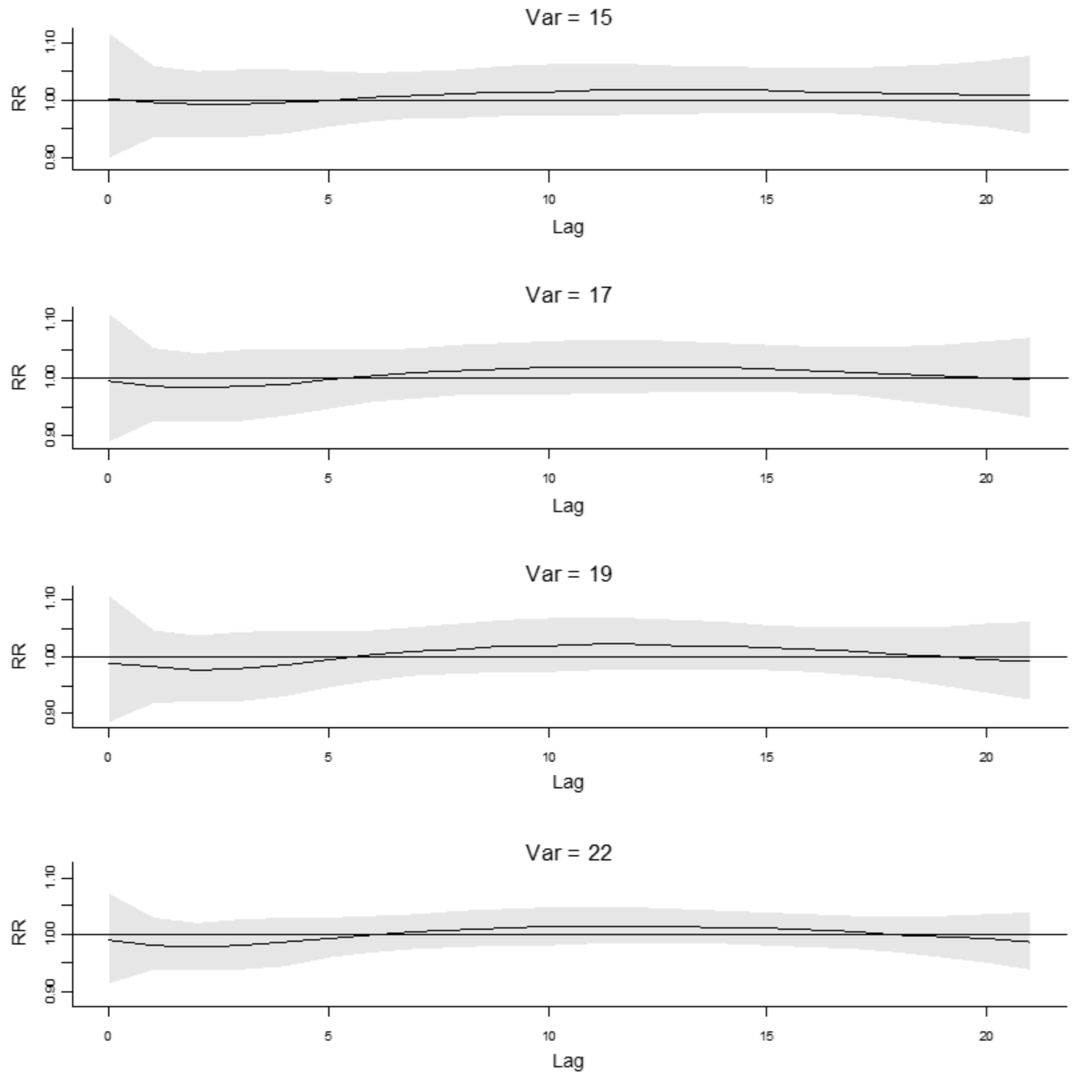

Figure S3 The temperature difference's (1st 5th 10th and 25th DTDmax) relative risk (include 95% C.I.) of AECOPD hospital admissions in the total population in different lag days

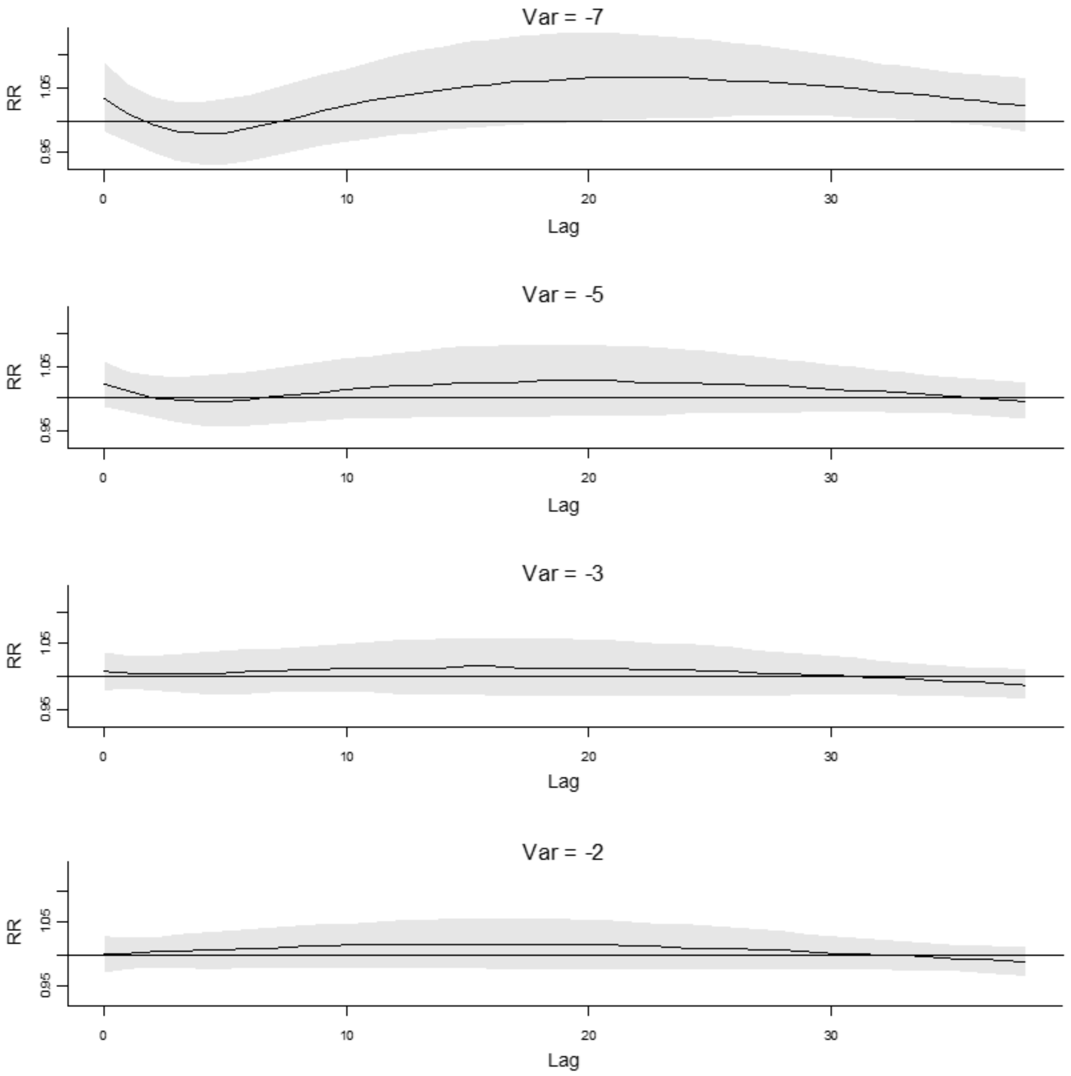

Figure S4 The temperature difference's (75th 90th 95th and 99th DTDmax) relative risk (include 95% C.I.) of AECOPD hospital admissions in the total population in different lag days

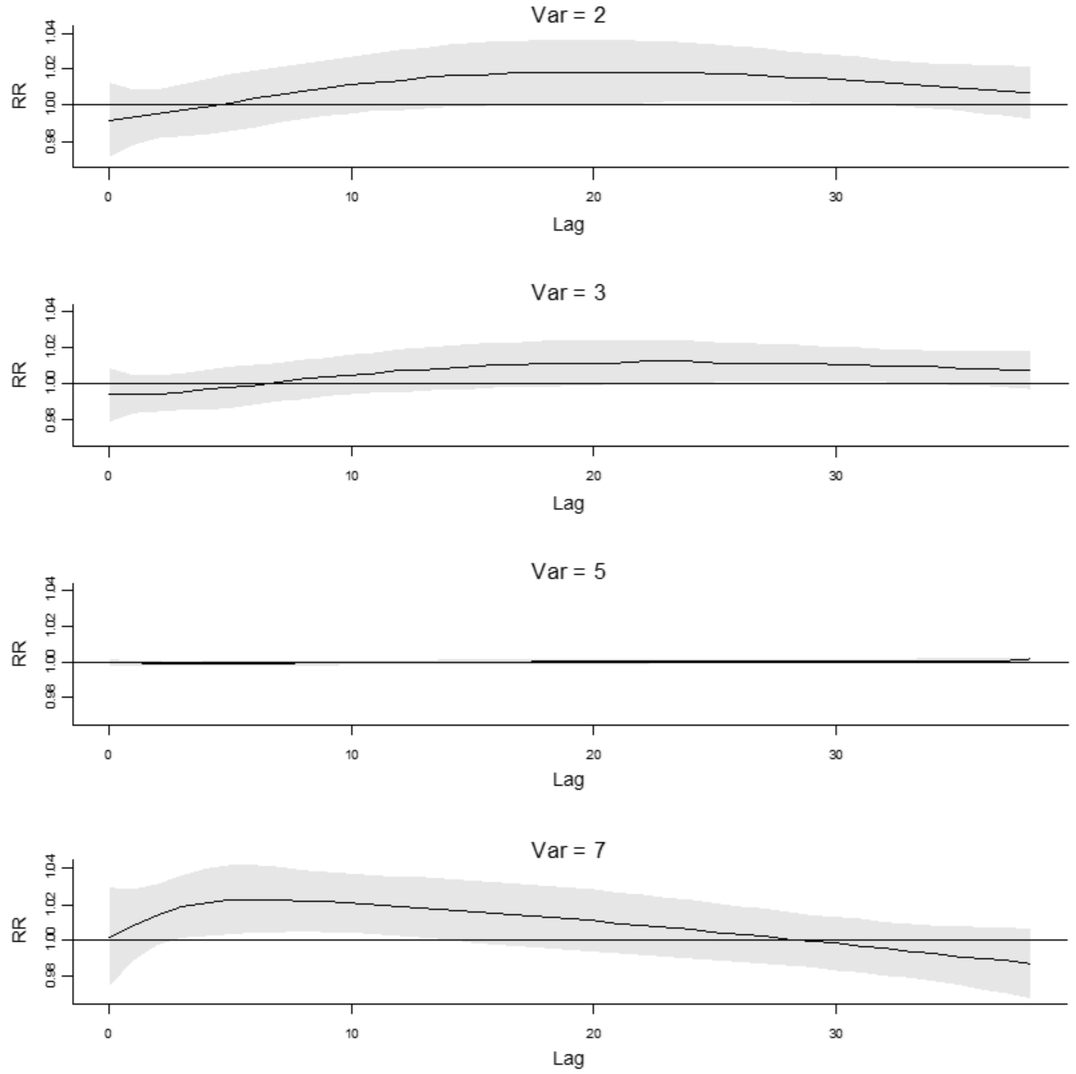

Figure S5 The temperature difference's (1st 5th 10th and 25th DTDmin) relative risk (include 95% C.I.) of AECOPD hospital admissions in the total population in different lag days

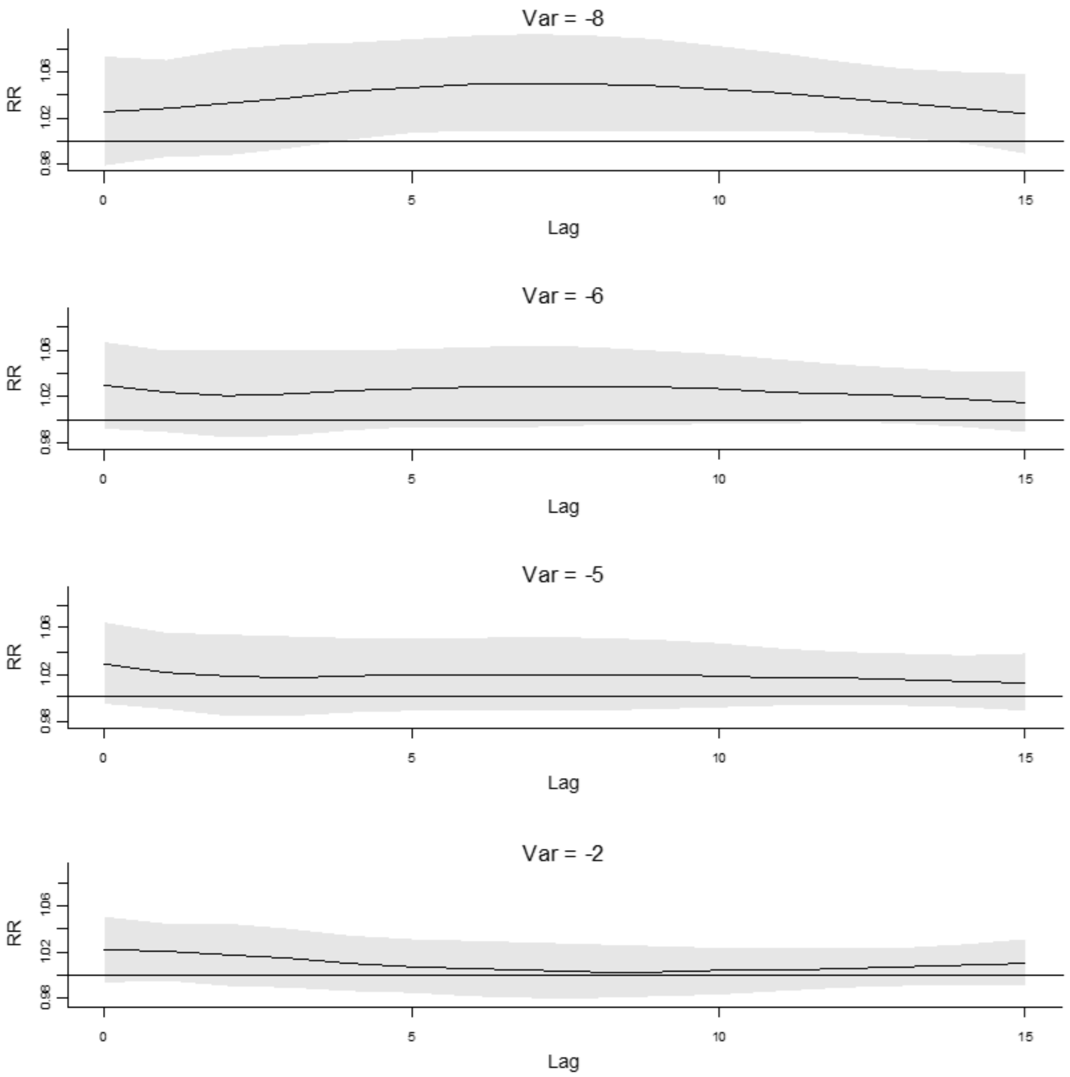

Figure S6 The temperature difference's (75th 90th 95th and 99th DTDmin) relative risk (include 95% C.I.) of AECOPD hospital admissions in the total population in different lag days

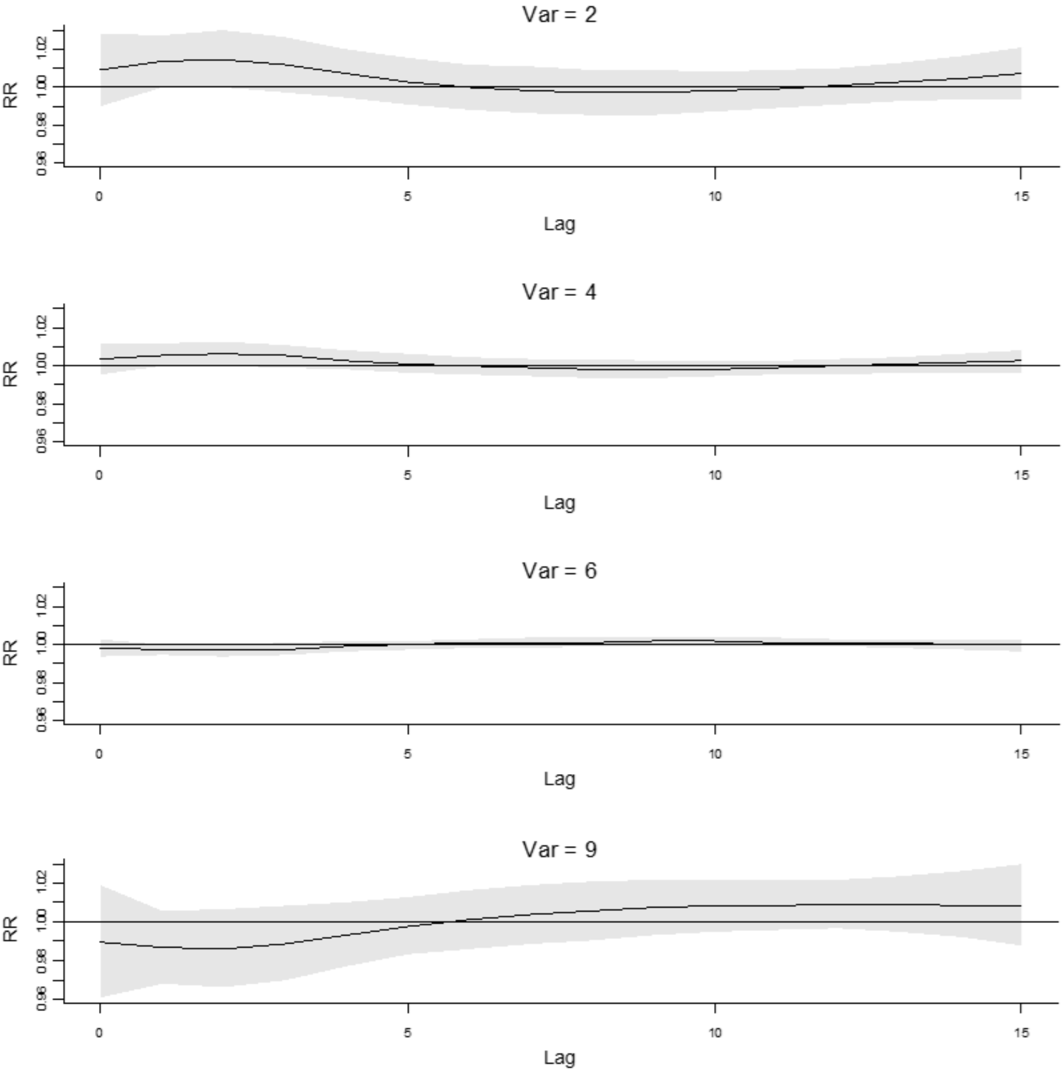

Figure S7 Association between the DTDmean and the 21 days cumulated relative risk (include 95% C.I.) of AECOPD hospital admissions in the total population in Beijing (df= 6 per year for *Time* and df=4 for RH, WS, AP, AQI)

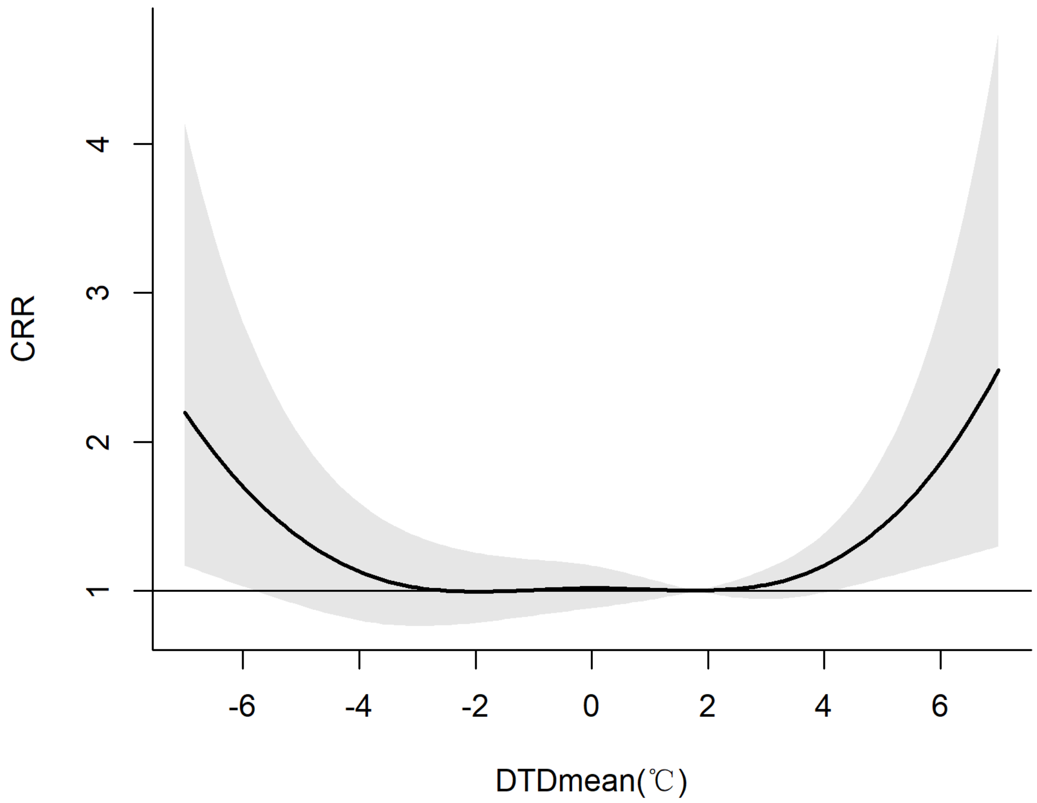

Figure S8 Association between the DTDmean and the 21 days cumulated relative risk (include 95% C.I.) of AECOPD hospital admissions in the total population in Beijing (df= 8 per year for *Time* and df=5 for RH, WS, AP, AQI)

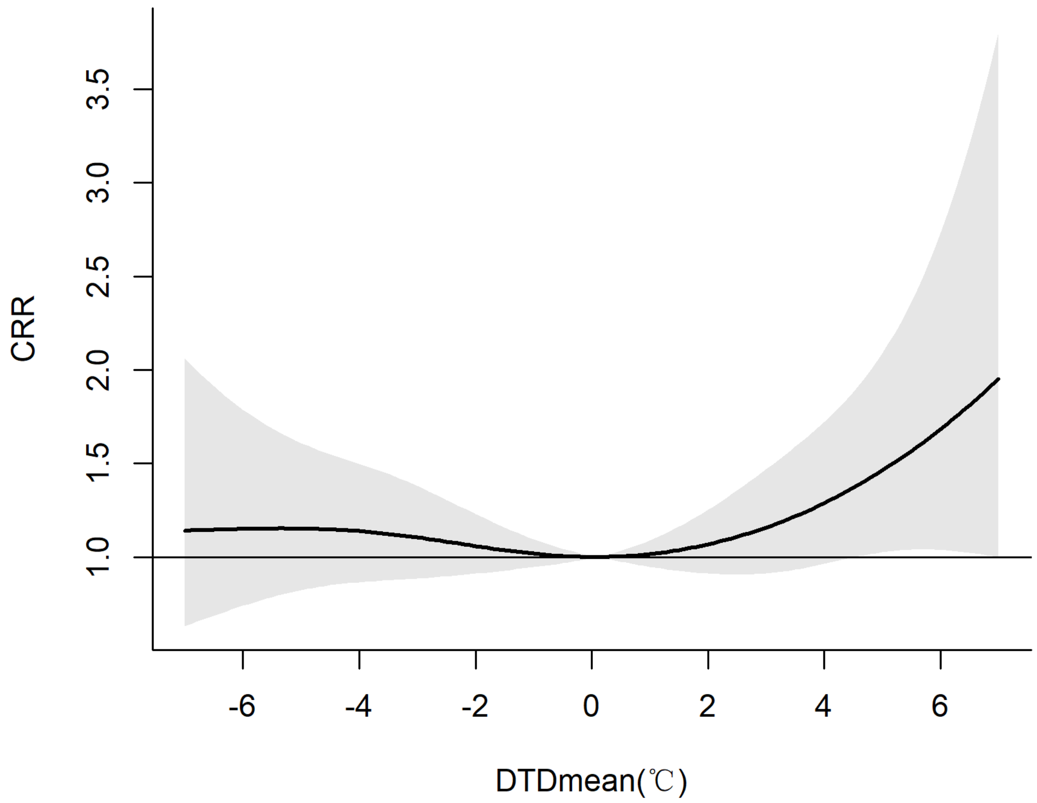

Supplement: Supplementary file 1 [file Data_Sheet_1.pdf]
